# Immersive virtual reality in orthopedic surgery as elective subject for medical students: First experiences in curricular teaching

**DOI:** 10.1007/s00132-024-04491-w

**Published:** 2024-04-04

**Authors:** Tobias Schöbel, Leonard Schuschke, Yasmin Youssef, Daisy Rotzoll, Jan Theopold, Georg Osterhoff

**Affiliations:** 1 Department of Orthopedics, Trauma and Plastic Surgery, University of Leipzig, Liebigstraße 20, 04103 Leipzig, Germany; 2 Skills and Simulation Centre LernKlinik Leipzig, Faculty of Medicine, University of Leipzig, Liebigstraße 23/25, 04103 Leipzig, Germany

**Keywords:** Augmented reality, Surgical education, Simulation training, Competency-based education, Medical education, Augmented Reality, Chirurgische Ausbildung, Simulationstraining, Kompetenzbasiertes Lernen, Medizinische Ausbildung

## Abstract

**Background:**

Virtual reality (VR) simulators have been introduced for skills training in various medical disciplines to create an approximately realistic environment without the risk of patient harm and have improved to more immersive VR (iVR) simulators at affordable costs. There is evidence that training on VR simulators improves technical skills but its use in orthopedic training programs and especially in curricular teaching sessions for medical students are currently not well established. The aim of this study was to describe the implementation of a VR operating theater as an elective course for undergraduate medical students and to evaluate its effect on student learning.

**Methods:**

An elective course for 12 students was implemented during the summer semester of 2023. Using Oculus Quest 2 headsets (Reality Labs, Meta Platforms, USA) and controllers and the PrecisionOS platform, they were able to train five different surgical procedures. The courses were accompanied by weekly topic discussions and instructional videos. Students were assigned to two groups: group VR vs. group non-VR. The groups were switched after 5 weeks. User feedback and performance development (theoretical and procedural surgical knowledge) after VR training were assessed using three questionnaires.

**Results:**

The students highly appreciated the implementation of VR training into their curriculum and 91% stated that they would opt for further VR training. All students stated that VR training improved their understanding of surgical procedures and that it should be obligatory in surgical training for undergraduate medical students. After 5 weeks of training, students in the VR group achieved significantly better results (100 out of maximum 180 points) than the non-VR group (70 points, *p* = 0.0495) in procedural surgical knowledge. After completion of the VR training the VR group achieved 106 points and the non-VR group 104 points (*p* = 0.8564). The procedural knowledge for non-VR group after 5 weeks significantly improved after VR training from 70 to 106 points (*p* = 0.0087).

**Conclusion:**

The iVR can be easily integrated into the curriculum of medical students and is highly appreciated by the participants. The iVR statistically improves the procedural knowledge of surgical steps compared to conventional teaching methods. Further implementation of iVR training in curricular teaching of medical students should be considered.

**Graphic abstract:**

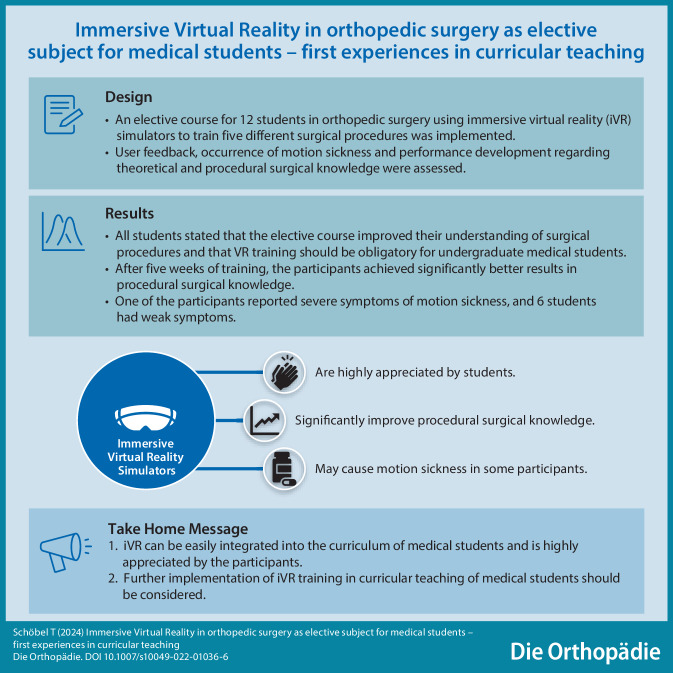

## Abstract (de)

**Einleitung:**

Simulatoren der virtuellen Realität (VR) wurden für das Training von Fertigkeiten in verschiedenen medizinischen Disziplinen eingeführt und haben sich technisch zu immersiven VR-Simulatoren (iVR) weiterentwickelt. Das Training an VR-Simulatoren verbessert die Fertigkeiten der Ausführenden, aber ihr Einsatz in der orthopädischen und unfallchirurgischen Ausbildung und vor allem in Lehrplänen für Medizinstudierende ist derzeit unzureichend etabliert. Das Ziel dieser Studie war es, Möglichkeiten zur Implementierung eines VR-Operationssaals als Wahlfach für Medizinstudierende zu beschreiben und den Effekt auf die theoretischen und praktischen Fähigkeiten der Studierenden auszuwerten.

**Methoden:**

Im Sommersemester 2023 wurde ein Wahlfach für 12 Studierende durchgeführt. Mithilfe von Oculus Quest 2‑Headsets und -Controllern sowie der PrecisionOS-Plattform konnten 5 verschiedene chirurgische Verfahren trainiert werden, diese wurden durch Vorträge und Lehrvideos begleitet. Die Studierenden wurden in zwei Gruppen eingeteilt: Gruppe VR vs. Gruppe non-VR. Die Gruppen wurden nach 5 Wochen getauscht. Das Benutzerfeedback und die Leistungsentwicklung (theoretisches und prozedurales Operationswissen) nach dem VR-Training wurden anhand von 3 Fragebögen bewertet.

**Ergebnisse:**

Die Studierenden lobten die Einführung der VR-Ausbildung in ihren Lehrplan außerordentlich und 91 % gaben an, weitere VR-Inhalte in der studentischen Ausbildung zu wünschen. Alle Studierenden gaben an, dass das VR-Training ihr Verständnis für chirurgische Verfahren verbessert hat und in der chirurgischen Ausbildung für Medizinstudenten obligatorisch sein sollte. Nach 5‑wöchigem Training erzielten die Studierenden der VR-Gruppe signifikant bessere Ergebnisse (100 von maximal 180 Punkten) als die non-VR-Gruppe (70 Punkte, *p* = 0,0495) im chirurgischen Verfahrenswissen und nach Abschluss des Trainings für beide Gruppen erreichte die VR-Gruppe 106 Punkte und die non-VR-Gruppe 104 Punkte (*p* = 0,8564). Das prozedurale Wissen der non-VR-Gruppe verbesserte sich nach 5 Wochen VR-Training signifikant von 70 auf 106 Punkte (*p* = 0,0087).

**Schlussfolgerung:**

iVR lässt sich leicht in den Lehrplan für Medizinstudierende integrieren und der Einsatz wird sehr gut evaluiert. iVR verbessert statistisch das Verfahrenswissen über chirurgische Schritte im Vergleich zu herkömmlichen Lehrmethoden. Eine weitere Implementierung von iVR-Training in die curriculare Ausbildung von Medizinstudierenden sollte in Betracht gezogen werden.

## Introduction

Surgical simulations are used for training skills in various disciplines to create an approximately realistic learning environment without the risk of patient harm [19]. The use of cadavers was a long-established gold standard training modality but there are limitations, such as high costs, limited accessibility, non-pathologic states, and ethical considerations. Therefore, new options for surgical training have been explored [14].

Virtual reality (VR) technologies are currently being used in orthopedic training simulators to increase surgical accuracy to improve outcomes and reduce complications [27–30]. Multiple studies have suggested that the use of VR during orthopedic residency programs improves surgical performance [9, 15, 22, 25] and VR simulators have been shown to be more effective in supporting the learning of residents than benchtop trainers or synthetic models [8, 15, 20]. Furthermore, the use of VR has been positively received by residents and associated with increased comfort in perceived surgical skills [1, 13].

Improvements in technology allowed the development of immersive virtual reality (iVR), which provides the advantages of conventional VR, whilst operating on low-cost, mobile, commercially available hardware [19, 24]. Mao et al. defined iVR as a fully virtual interactive simulation with a 3-dimensional (3D) environment projected onto a head-mounted display (HMD), allowing for a 360° visual immersion and real-time manipulation of virtual items. Other sensory modalities (e.g., haptic, auditory) were not required to meet this criterion [19]. Although there is extensive evidence that training on VR simulators improves technical skills, the use in orthopedic training programs lags behind other surgical specialties [1]. Training simulators are only available in a few residency programs [29] and only one study showed usage of VR in curricular education in orthopedic and trauma surgery for medical students [10].

Data are lacking on the experience of iVR use in the curricular training of medical students. The aim of this study was to describe the framework conditions and implementation for the use of VR techniques in the teaching of medical students in orthopedic surgery and to obtain user feedback of medical students on this educational method. In addition, the effects of iVR on students’ theoretical and procedural learning were evaluated.

## Methods

### Curricular implementation

In the medical curriculum at Leipzig University, all medical students have to complete at least one elective course in each of their preclinical and clinical training. This elective course consists of a minimum of 27 teaching units of 45 min and must completed by an examination.

The elective “Virtual Reality operating room (OR) course in Orthopedics and Trauma Surgery” was introduced in the summer semester 2023 for 12 students in 2 courses (6 male and 6 female students; average age 25.1 years) from 10 May 2023 to 12 July 2023. The students were randomly assigned to two groups: group VR (*n* = 6) and group non-VR (*n* = 6). A different surgical procedure was taught each week as follows:

1. Intramedullary nail fixation for a proximal femoral fracture.
2. Total knee arthroplasty.
3. Total shoulder arthroplasty.
4. Dorsal stabilization of a spinal fracture.
5. Anterior cruciate ligament (ACL) reconstruction.

**Fig. 1:**
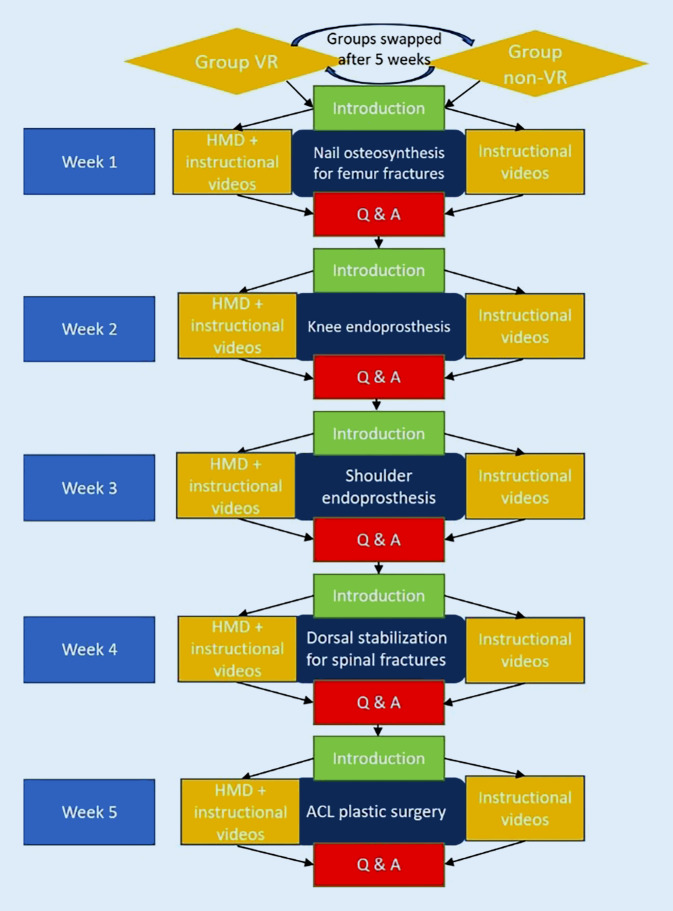
Flowchart of the curriculum. Both groups were switched after 5 weeks

Each surgical procedure was explained to both groups in a 20 min presentation by an academic surgeon specialized in the procedure, followed by a question and answer (Q&A) seminar. In addition, all students were provided with two instructional videos about the procedure. Each student in the VR group received a stand-alone head-mounted display (HMD) with the corresponding software for a total of 5 weeks and had to work through the operations independently. The two groups were switched after 5 weeks (Fig. 1). Apart from the first introductory unit and the unit after 5 weeks, where the HMDs were exchanged, all teaching units took place digitally. Device maintenance and output, management of software updates and the initial device instructions were managed with the support of the faculty’s Skills and Simulation Center.

### Technical equipment

Six Oculus Quest 2 headsets (Reality Labs, Meta Platforms, USA) and corresponding controllers were used as a stand-alone head-mounted display. To ensure anonymization, preconfigured meta-accounts were provided for all six HMDs. As software PrecisionOS Platform Version 3.0 (PrecisionOS Technologies, Canada) was provided for each HMD. Each student received a technical introduction at the beginning of the course. The online teaching units took place via BigBlueButton (BigBlueButton Inc., Ontario, Canada) to allow student-teacher exchange. Instructional videos were provided via Vumedi (https://www.VuMedi.com) and Youtube (https://www.youtube.com).

### Data acquisition

The students were asked to complete a total of two written assessments and a final questionnaire.

The first and second written assessments were completed on paper after 5 and 10 weeks, respectively. Each written assessment had three aspects for each of the course weeks:

- Theoretical knowledge: answering a theoretical question (e.g., indications for intramedullary nail osteosynthesis in femur, maximum 1 point).
- Procedural knowledge: putting surgical steps in the correct order (maximum 6 points).
- Quality of care assessment: rating three different postoperative radiographs by quality of care (perfect result, poor result not requiring revision, poor result requiring revision; maximum 3 points).

The scoring was accumulated for each student group (*n* = 6) and the 5 courses to a maximum of 30 points in theoretical knowledge, a maximum of 180 points for procedural knowledge and a maximum of 90 points in quality of care assessment for each group.

**Fig. 2:**
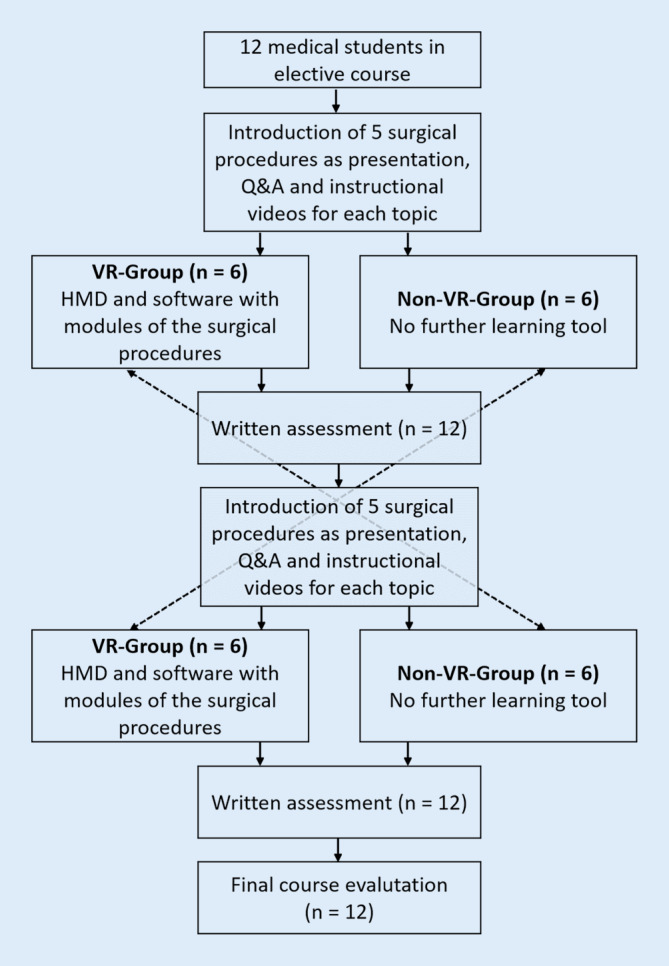
Flowchart of assessments and final evaluation

With this setup it was possible to compare the effects of the VR training compared to the lectures and instructional videos alone (group VR after 5 weeks vs. group non-VR after 5 weeks), as well as the improvements after VR training (group non-VR after 5 weeks and group VR after 10 weeks) and possible differences between both groups after 10 weeks (group VR after 10 weeks vs. group non-VR after 10 weeks). After 10 weeks, a course evaluation was conducted consisting of 25 items. This primarily covered user satisfaction, user behavior, technical problems with hardware or software, and the occurrence of motion sickness. In addition, free text questions were asked about the potential for improving the software modules (Fig. 2).

### Statistical analysis

Statistical analysis was performed using SPSS (IBM Corp. released 2016, IBM SPSS Statistics for Windows, Version 24.0, Armonk, NY, USA). Data from the first and second assessments were compared using the Mann-Whitney U‑test or the Fishers’ exact test. Significance was set at *p* < 0.05.

### Data protection

No patient data were published in the lectures. All students had given informed consent to the use of anonymized data as part of the survey. To ensure anonymization, preconfigured meta-accounts were provided.

## Results

### Effects of VR training

**Fig. 3:**
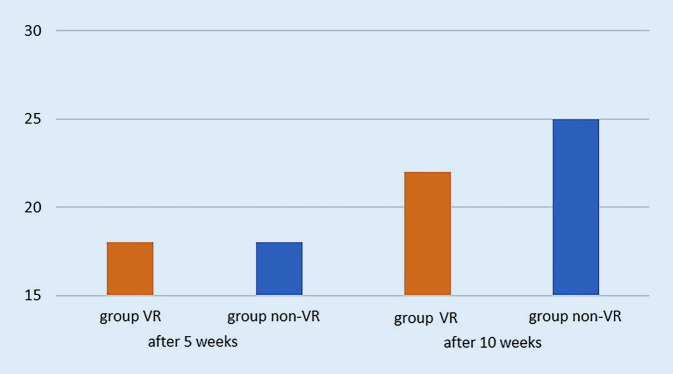
Total score of theoretical knowledge with a maximum of 30 points (on the y-axis) after 5 and 10 weeks for both groups. There was no statistically significant difference

For theoretical knowledge both groups achieved a total of 18 out of 30 points after 5 weeks (*p* = 1.0). After 10 weeks group VR yielded 22 and group non-VR reached 25 of 30 points (*p* = 0.5321) (Fig. 3).

**Fig. 4:**
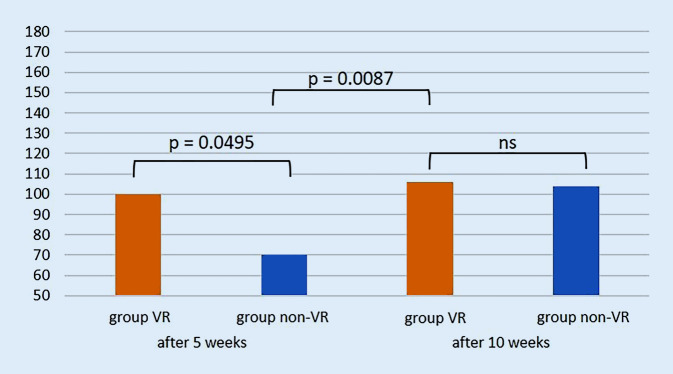
Total score of procedural knowledge of the surgical steps with a maximum of 180 points (on the y-axis) after 5 and 10 weeks for both groups

**Fig. 5:**
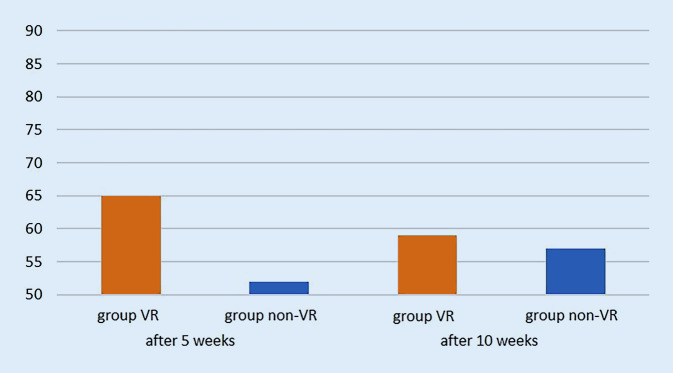
Total score of assessment of postoperative radiographs with a maximum of 90 points (on the y-axis) after 5 and 10 weeks for both groups. There was no statistically significant difference

After 5 weeks of training, students in the VR group achieved significantly better results (100 of maximum 180 points) than group non-VR (70 points, *p* = 0.0495) in procedural surgical knowledge. After completion of the VR training for both groups, the VR group achieved 106 points and group non-VR 104 points (*p* = 0.8564). The procedural knowledge for group non-VR after 5 weeks significantly improved after VR training from 70 to 106 points (*p* = 0.0087; Fig. 4). For assessment of the postoperative radiographs, group VR achieved 65 of 90 points, whereas group non-VR achieved 52 points after 5 weeks (*p* = 0.1684). After 10 weeks, group VR achieved 59 points compared to 57 points in group non-VR (*p* = 0.8361). There was no statistically significant improvement for assessment of the postoperative radiographs after VR training (*p* = 0.48, Fig. 5).

### User feedback

The online evaluation was answered by 11 of the 12 participating students (92%). Of the 25 items there were 10 questions about user satisfaction with hardware and software for an average score on a scale from 1 (worst) to 10 (best). These evaluations are represented in Table 1.

**Table 1:** Table 1 represents the evaluation of the user satisfaction with hardware and software for an average score on a scale from 1 (worst) to 10 (best)

User satisfaction with hardware and software, 1 (worst) to 10 (best)	Average score
How likely would you recommend this VR tool to a fellow student?	8.45
How satisfied were you with the PrecisionOS virtual reality training modules?	7.09
How satisfied were you with the selection of different modules for your training?	8.45
How would you rate the product in general?	8.2
How easy was it to use the PrecisionOS training modules?	6.9
How realistic were the PrecisionOS training modules?	6.3
How would you rate the PrecisionOS training modules overall?	8.1
How comfortable was it to wear the VR goggles?	6.1
How easy was it to use the Meta Quest VR headset?	8.1
How easy was it to handle the headset using the controllers?	8.7

**Fig. 6:**
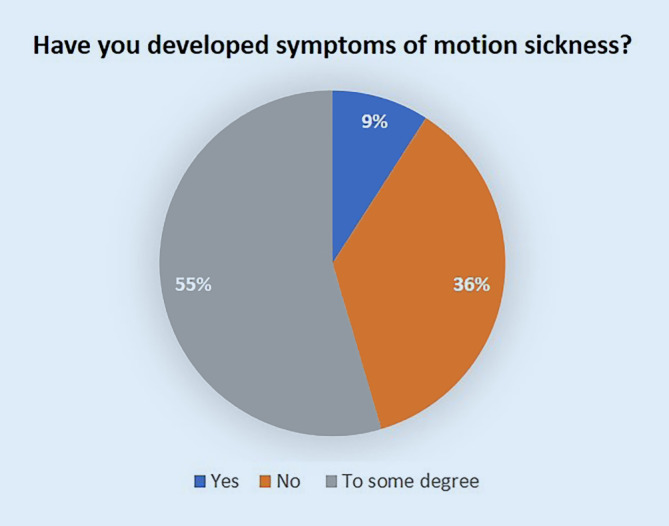
Occurence of motion sickness

In both groups most students stated that they watched 50% or less of the instructional videos after 5 and 10 weeks. When asked if the students would use the iVR tool again, 91% answered positively. All students stated that the iVR training had improved their understanding of surgical procedures and 67% stated that it improved their surgical skills. All students expressed that they would prefer more iVR in surgical training in their studies and 73% of the participants believed that this kind of training method can be used to improve operative outcomes (Table 2). Most students (91%) stated that they have used the tool at least once per week. On the technical side, 55% of the students stated that the explanations of the PrecisionOS training modules were understandable and easy to follow. The difficulty of the modules was rated as exactly right by 91% of participants. When asked if the students missed any training modules, 27% stated yes and suggested to include more modules for fracture osteosynthesis and hip arthroplasty. Occasional technical complications with the HMD were reported by 82% of the participants (Table 3). One of the students reported severe symptoms of motion sickness and six students had weak symptoms (Fig. 6).

**Table 2:** User feedback of subjective impact on surgical skills in the final evaluation

Item	Yes	No	Not sure
If you had the opportunity, would you use the tool again?	10	1	x
Did the VR training improve your understanding of surgical procedures?	11	0	x
Has the VR training improved your surgical skills?	6	3	2
Would you prefer to use more VR in surgical training?	11	0	0
Do you believe that this training method can improve surgical results?	8	3	0

*x* no selectable answer option

**Table 3:** User feedback of the training modules in final examination

Item			
Have you already used VR training modules other than PrecisionOS?	*Yes*	*No*	*Not sure*
	1	8	2
Did you miss any training modules in the selection?	3	9	x
The explanations of the PrecisionOS training modules were understandable and easy to follow	*Yes*	*No*	*Partially*
	6	0	5
How would you rate the difficulty of the PrecisionOS training modules you have completed?	*Exactly right*	*Too easy*	*Too hard*
	9	1	0
Did you have any technical complications with the VR headset?	*Yes, occasionally*	*No*	*Yes, many*
	9	2	0

*x* no selectable answer option

When asked what was positive about the modules, the students praised the easy and intuitive handling, the possibility to depict the anatomical structures outside of the operative site and most frequently the structured guiding for the operations available. When asked which aspects could be improved the students mostly criticized technical bugs, the sometimes imprecise handling and especially the lack of a haptic feedback.

## Discussion

The aim of this study was to describe the implementation of an iVR operating theater as an elective course for medical students and to evaluate its effect on the students’ theoretical and practical learning. The use of VR stimulation in orthopedic training programs is less common than in other surgical specialties [1] and training simulators are rarely available for orthopedic residents [29]. Regarding medical students or surgical interns, other disciplines show a plethora of VR tools in medical education for non-residents [5]. In orthopedic surgery, only one study reported the usage of VR training for medical students, introducing students to an intensive care unit and prehospital care [10]. Atli et al. described an iVR application for neurosurgical education for medical students with almost the same setup as the presented study: 12 students were included in an elective course in neurosurgery for 1 year, including an iVR-based learning platform [3]. As shown in both studies, the implementation of VR training for medical students is feasible for a low number of students as an elective course; however, the technical infrastructure and access to hardware and software may be a bottleneck when more students are involved. The approximate cost of iVR platforms varies between US$ 1500 and US$ 8000, depending on the institutional licensing agreements [6, 11, 16], which is estimated to be up to 34.1 times more cost-effective than traditional training methods [18]. Nevertheless, factors such as device maintenance and output, management of software problems and updates as well as device instructions must be considered and were only manageable in the setup described with the support of the faculty’s Skills and Simulation Center.

In the evaluation of Atli et al. all students agreed that utilizing iVR helped them gain a deeper understanding of neuroanatomy and neurosurgery and 69% claimed to have a better understanding of neurosurgical skills. All of the students evaluated the course to be a valuable learning experience and iVR a useful learning tool [3]. These findings are in line with the results of the present study and indicate favorable results and high acceptance of iVR in medical education especially in surgical disciplines. The authors concluded that iVR training may improve confidence and therefore could contribute to improved participation during surgical rotations and better understanding for operative procedures [3, 19], which is also confirmed by the results of the present study. The students’ evaluations in both studies are in good agreement with the largely positive user feedback of surgical residents evaluating the usability of different iVR simulators [4, 11, 18]. In a systematic review, Mao et al. stated that residents enjoyed iVR training significantly more than theoretical training and tended to perceive iVR training to be more useful than theoretical training in general. This was especially the case for novice surgeons [19], which are probably closer to students in terms of their level of training than specialized surgeons.

In the present study, students stated to be very satisfied with the iVR training and that they would recommend the tool to a fellow student. This confirms findings from other studies that reported iVR training to be realistic, usable and useful in surgical training [4]. The main point of criticism was the frequently insufficient tactile feedback in the applications. This flaw was widely reported in other investigations [2, 3, 11, 16, 21]. In 2008 Praamsma et al. showed that an auditory stimulus using bone drilling sounds may act in a same way as haptics for expert surgeons [23]; however, the iVR tool used in the present study emitted a realistic auditory stimulus (e.g., bone drilling) to supplement tactile feedback and despite this was criticized by the users to be insufficient. While this may be explained by the lack of experience in medical students, the main point of criticism confirms that improving haptic and auditory feedback may further improve the efficacy of iVR training [19]. A second consideration of using iVR training is the occurrence of motion sickness, caused by a mismatch between the sensory systems by using HMDs and resulting in nausea and ultimately vomiting [10]. Motion sickness was reported by 23% of the participants by Holla et al. [10], whereas Barré et al. reported slight to moderate symptoms of motion sickness in 30% of their participants [4]. In the present investigation, one of the participants reported severe symptoms of motion sickness, while 55% reported weaker symptoms. This wide variety may be explained by differences in the HMD used and different levels of practice with iVR training by the participants, as postural discomfort has shown to decrease with increased practice [4]. As a solution for participants showing symptoms, chewing gum during VR training is known to reduce symptoms of motion sickness [12] and a study showed that less nausea is reported when the HMD can be adjusted to properly fit the interpupillary distance [10, 26].

The positive effects of VR training in surgery were summarized in a recent systematic review by Mao et al. who found 11 controlled trials comparing iVR training with non-VR training and in 2 of these trials the control group was taught with written material plus video explanations or video material only, which is comparable to our test setup [16, 17, 19]. Mao et al. found that groups that were trained with iVR performed 18–43% faster in surgical procedures as compared to control groups without iVR training, demonstrated greater postintervention scores on procedural checklists and greater implant placement accuracy [19]. Lohre et al. evaluated whether iVR improved learning effectiveness in 18 senior orthopedic surgery residents with comparable surgical experience during a single training course. The residents were randomized into two groups: one group received training on the PrecisionOS platform version 3.0 (PrecisionOS Technology) and the control group received training using a surgical video of a reverse shoulder arthroplasty (RSA) with augmented baseplate. Following the intervention, each participant was tested on theoretical knowledge about the procedure and had to perform RSA in a cadaveric model. The iVR-trained group significantly outperformed the non-VR group in the objective structured assessment of technical skills (OSATS) and the verbal questioning scores [17]. This is comparable to the results of the presented study to some degree as the students in the VR group also showed statistically significant higher procedural knowledge compared to the non-VR group and statistically improved in their final score after 5 weeks of training. Logishetty et al. compared a total of 24 surgical trainees: 12 completed a 6-week VR training program, while the non-VR group received only conventional preparatory materials. Afterwards all 24 trainees had to perform a cadaveric total hip arthroplasty (THA) and their technical and non-technical surgical performance was measured by a THA-specific procedure-based assessment. The VR-trained surgeons completed 33% more key steps than controls, were 12% more accurate in component orientation and were 18% faster than the non-VR group, showing higher procedural knowledge and psychomotor skills [16]. Furthermore, Blumstein et al. compared 20 students with and without VR training in intramedullary nail fixation of tibial shaft fractures. The VR-trained students significantly outperformed the other conventionally trained group on the procedure-specific checklist and showed significantly higher knowledge of the surgical instruments [7]. Again, all these findings are supported by the presented data, showing significantly higher procedural knowledge after iVR training.

This study has some limitations: (1) the small group sample of *n* = 12 medical students only allows limited conclusions regarding the influence of VR training on theoretical and procedural knowledge gain by the students. (2) The outcomes where not validated in objective scores such as OSATS or procedure-based assessment (PBA). (3) The evaluation of motion sickness was insufficiently structured and did not allow differentiation between the exact symptoms experienced by the students.

## Conclusion

Immersive virtual reality can be easily implemented into the curriculum of medical students and is highly appreciated by the participants. Students prefer more iVR in surgical training in their medical education and state that it improves their understanding of surgical skills. iVR statistically improves the procedural knowledge of surgical steps compared to instructional videos.

## Data Availability

The datasets used and/or analyzed during this study are available from the corresponding author upon reasonable request.

## Abbreviations

VR: virtual reality
iVR: immersive virtual reality
3D: 3-dimensional
HMD: head-mounted-display
ACL: anterioir cruciate ligament
Q&A: Question and Answer
RSA: reverse shoulder arthroplasty
OSATS: objective structured assessment of technical skills
THA: total hip arthroplasty
PBA: procedure-based assessment
OR: operating room
